# Analysis of the genomic landscapes of Barbadian and Nigerian women with triple negative breast cancer

**DOI:** 10.1007/s10552-022-01574-x

**Published:** 2022-04-06

**Authors:** Shawn M. Hercules, Xiyu Liu, Blessing B. I. Bassey-Archibong, Desiree H. A. Skeete, Suzanne Smith Connell, Adetola Daramola, Adekunbiola A. Banjo, Godwin Ebughe, Thomas Agan, Ima-Obong Ekanem, Joe Udosen, Christopher Obiorah, Aaron C. Ojule, Michael A. Misauno, Ayuba M. Dauda, Ejike C. Egbujo, Jevon C. Hercules, Amna Ansari, Ian Brain, Christine MacColl, Yili Xu, Yuxin Jin, Sharon Chang, John D. Carpten, André Bédard, Greg R. Pond, Kim R. M. Blenman, Zarko Manojlovic, Juliet M. Daniel

**Affiliations:** 1 Department of Biology, McMaster University, Hamilton, ON Canada; 2 African Caribbean Cancer Consortium, Philadelphia, PA USA; 3 Department of Translational Genomics, Keck School of Medicine, University of Southern California, Los Angeles, CA USA; 4 Stem Cell and Cancer Research Institute (SCC-RI), McMaster University, Hamilton, ON Canada; 5 Faculty of Medical Sciences, University of the West Indies at Cave Hill, Bridgetown, Barbados; 6 Department of Pathology, Queen Elizabeth Hospital, Bridgetown, Barbados; 7 Department of Radiation Oncology, Queen Elizabeth Hospital, Bridgetown, Barbados; 8 Present Address: Cancer Specialists Inc, Bridgetown, Barbados; 9 Department of Anatomic and Molecular Pathology, Lagos University Teaching Hospital, Lagos, Nigeria; 10 Department of Pathology, University of Calabar Teaching Hospital, Calabar, Nigeria; 11 Department of Obstetrics & Gynaecology, College of Medical Sciences, University of Calabar Teaching Hospital, Calabar, Nigeria; 12 Department of Pathology, College of Medical Sciences, University of Calabar Teaching Hospital, Calabar, Nigeria; 13 Division of General and Breast Surgery, University of Calabar Teaching Hospital, Calabar, Nigeria; 14 Department of Anatomical Pathology, University of Port Harcourt Teaching Hospital, Port Harcourt, Nigeria; 15 Department of Chemical Pathology, University of Port Harcourt Teaching Hospital, Port Harcourt, Nigeria; 16 Department of Surgery, Jos University Teaching Hospital, Jos, Nigeria; 17 Department of Pathology, Jos University Teaching Hospital, Jos, Nigeria; 18 Meena Histopathology and Cytology Laboratory, Jos, Nigeria; 19 Department of Mathematics, University of the West Indies at Mona, Kingston, Jamaica; 20 Present Address: Wang Yanan Institute for Studies in Economics, Xiamen University, Xiamen, China; 21 Department of Pathology and Molecular Medicine, McMaster University, Hamilton, ON Canada; 22 Department of Health Research Methods, Evidence, and Impact, McMaster University, Hamilton, ON Canada; 23 Department of Oncology, McMaster University, Hamilton, ON Canada; 24 Department of Internal Medicine, Section of Medical Oncology, Yale Cancer Center, School of Medicine, New Haven, CT USA; 25 Department of Computer Science, School of Engineering and Applied Science, Yale University, New Haven, CT USA

**Keywords:** Triple negative breast cancer, Women of African ancestry, Whole exome sequencing, Genomics

## Abstract

**Purpose:**

Triple negative breast cancer (TNBC) is an aggressive breast cancer subtype that disproportionately affects women of African ancestry (WAA) and is often associated with poor survival. Although there is a high prevalence of TNBC across West Africa and in women of the African diaspora, there has been no comprehensive genomics study to investigate the mutational profile of ancestrally related women across the Caribbean and West Africa.

**Methods:**

This multisite cross-sectional study used 31 formalin-fixed paraffin-embedded (FFPE) samples from Barbadian and Nigerian TNBC participants. High-resolution whole exome sequencing (WES) was performed on the Barbadian and Nigerian TNBC samples to identify their mutational profiles and comparisons were made to African American, European American and Asian American sequencing data obtained from The Cancer Genome Atlas (TCGA). Whole exome sequencing was conducted on tumors with an average of 382 × coverage and 4335 × coverage for pooled germline non-tumor samples.

**Results:**

Variants detected at high frequency in our WAA cohorts were found in the following genes *NBPF12*, *PLIN4*, *TP53* and *BRCA1*. In the TCGA TNBC cases, these genes had a lower mutation rate, except for *TP53* (32% in our cohort; 63% in TCGA-African American; 67% in TCGA-European American; 63% in TCGA-Asian). For all altered genes, there were no differences in frequency of mutations between WAA TNBC groups including the TCGA-African American cohort. For copy number variants, high frequency alterations were observed in *PIK3CA, TP53, FGFR2* and *HIF1AN* genes.

**Conclusion:**

This study provides novel insights into the underlying genomic alterations in WAA TNBC samples and shines light on the importance of inclusion of under-represented populations in cancer genomics and biomarker studies.

**Supplementary Information:**

The online version contains supplementary material available at 10.1007/s10552-022-01574-x.

## Introduction

Breast cancer (BCa) is currently the second leading cause of cancer-related deaths in women worldwide [1] and is routinely categorized into different subtypes based on the amplification of human epidermal growth factor receptor 2 (HER2) and expression of estrogen receptor (ER) and progesterone receptor (PR) [2]. Tumors that lack expression for these three receptors are classified as **t**riple **n**egative **b**reast **c**ancer (TNBC). These tumors are typically more aggressive with advanced grade and stage at diagnosis and limited targeted therapies due to the absence of HER2, ER and PR [3]. Mounting evidence indicates a higher prevalence of TNBC in West African women and **w**omen of African **a**ncestry (WAA) in the Caribbean (~ 25% in Barbados), the USA (~ 22%) and the UK (~ 22%) compared to non-Hispanic White women (11%) [4–6]. Previous studies have noted variable TNBC estimates across the African continent where West African populations have been shown to have higher TNBC estimates compared to North, East and Southern African regions [7]. The reasons for these disparities are currently unknown; however, recent studies allude to an intricate interplay of environmental and genetic risk factors [8–10].

Due to the aggressive nature of TNBC, there has been an increased interest in investigating molecular biomarkers that could be relevant for therapeutics, diagnostics, and prognostics. Recently, it was found that a subset of TNBC patients with deleterious *BRCA1/2* germline mutations responded significantly better to carboplatin (platinum-based therapy) than docetaxel (taxane-based therapy) [11]. In addition to the clinical utility of germline variants, the identification of novel somatic “driver” mutations has been shown to play critical roles in the development of targeted therapies in breast and other solid tumors [12, 13]. Therefore, identification of molecular targets and subsequent development of targeted therapies will be of great importance in improving the overall survival rates of TNBC patients.

Large cancer genomics databases, such as The Cancer Genome Atlas (TCGA), have been useful in understanding the genomics landscape of a variety of tumors. However, to date, most of the TCGA breast cancer biospecimens are from women of European ancestry (~ 80%) despite higher TNBC prevalence in women of African and Hispanic ancestry [14, 15]. Due to the low percentage of African ancestry cases within the TCGA and other similar repositories, researchers have embarked on conducting independent sequencing studies on these populations to explore and understand their unique genomics landscapes [16–21]. Research consortiums such as the International Consortium for the Study of Breast Cancer Subtypes (ICSBCS), the African Caribbean Cancer Consortium (AC3), the African Organization for Research and Training in Cancer (AORTIC), and work done by the Nigerian Breast Cancer Study have begun to shed significant light on potential biomarkers among diverse populations of African ancestry [17, 18, 21–23]. Herein, we have conducted **w**hole **e**xome **s**equencing (WES) of ancestrally related WAA with TNBC in Nigeria and Barbados to further understand the genomics landscape of these groups. Previous genetic association studies have estimated a high percentage of West African (specifically Nigerian) ancestry in Barbadian cohorts with 80%-90% West African ancestry and admixture with European ancestry, thus the rationale for these two groups [24–26]. Our findings were compared with the genomic signature of TNBC cases of African American (TCGA-AA) and European American (TCGA-EA) within the TCGA database.

## Methods

### Patient population

Formalin-fixed paraffin-embedded (FFPE) specimens with corresponding clinical data were collected from the Pathology Department at the Queen Elizabeth Hospital (QEH) in Barbados, Lagos University Teaching Hospital (LUTH), University of Calabar Teaching Hospital (UCTH), University of Port Harcourt Teaching Hospital (UPTH), and Jos University Teaching Hospital (JUTH) in Nigeria. Fifty-nine FFPE TNBC samples were selected at random for DNA extraction and WES, however only 31 samples passed quality control. Protocols for specimen collection as outlined within the respective institutional review boards were adhered to and patients consented to give their samples. The study was approved by the institutional review boards at McMaster University, the University of the West Indies—Cave Hill, the QEH, LUTH, UCTH, UPTH and JUTH. Tissues were assessed for ER, PR and HER2 via immunohistochemistry (IHC) at their respective institutions and BCa subtype status was further confirmed at McMaster University in Canada. The Allred algorithm [27] was used to calculate the scores.

### Pathologic assessment and DNA isolation

Hematoxylin and eosin (H&E) stained slides were made from FFPE samples for pathological interrogation of tumor enrichment. 10 µm FFPE tissue sections on slides were scraped and placed in NAVY RINO tubes (Next Advance, Troy, NY) with stainless steel beads and 160 µL of deparaffinization solution (Qiagen, Hilden, Germany). Samples were homogenized using the Bullet Blender (Next Advance) for 5 min at speed 12. Samples were incubated for 3 min at 56 °C and processed according to the manufacturer’s instructions. DNA was isolated from pathologically assessed tumor-enriched regions and uninvolved “normal” sections.

### Whole exome sequencing

Quality and quantity of DNA was measured using the Genomic DNA Screen Tape Assay (Agilent Technologies, Santa Clara, CA) and Qubit. The concentration of genomic DNA (gDNA) larger than 200 bp was then calculated, and at least 200 ng of DNA greater than 200 bp was sheared in 50 µL of nuclease-free water with the Covaris E220 using the 96 microTUBE Plate (Covaris, Woburn, MA). The library was prepared using KAPA Hyper Prep Kit (Roche, Basel, Switzerland) and 100–500 ng of sheared DNA according to the manufacturer’s instructions. Individual tumor adapter-ligated libraries were enriched into the exome capture reaction, and for germline each adapter-ligated library was pooled before proceeding to capture using Agilent’s SureSelect Human All Exon V6 + custom probes capture library kit [28]. Samples that had successful libraries created were then sequenced on Illumina MiSeq technology for quality control to assess the ability of the libraries to be sequenced. Subsequently, each library was pooled and sequenced on Illumina’s NovaSeq 6000 (Illumina, San Diego, CA) using 300 cycle kit. Raw FASTQs were generated using the industry standard BCL2FASTQ v1.8.4

### Primary informatics methods

Whole exome sequences were aligned by BWA (v0.7.17) to GRCh38. Quality score errors were detected by GATK's Base Recalibrator (v4.0.10.1). Picard Tools (v1.128) was used to merge aligned BAMs and mark duplicate reads. Germline Variant Call Format (VCF) of BAM were obtained by GATK's Haplotype Caller using GATK best practices, Samtools MPileUp together with BCFtools (v1.2), and Freebayes (v1.1.0–6-gf069ec6). Somatic variant calling was performed by MuTect2 [29] to ensure compatible comparison between with TCGA. MuTect2 somatic variant calling files (VCF) for each patient in this study were converted to MAF files using the vcf2maf v1.6.19 tool [30]. Data from TCGA were downloaded from: https://portal.gdc.cancer.gov/projects/TCGA-BRCA. The TNBC subtypes were extracted and divided into self-reported Caucasian/European American (EA) and African American (AA) race. Non-silent variants reported were validated visually using IGV (v2.7.2) [31].

### Downstream bioinformatics methods

Gene frequencies in our WES data were performed by Unified Optimal Sequence Kernal Association testing in R. Visualization of somatic variants was performed using maftools (v2.6.05) [32] and R packages pheatmap (v1.0.12), ggplot2 (v3.3.3), VennDiagram (v1.6.20), and ggrepel (v0.9.1). Mutation signature from WES data were computed using Mutational Signature in Cancer (MuSiCa) [33]. Copy number analysis was performed utilizing Nexus Copy Number v10 (Biodiscovery) and focal analysis was performed by GISTIC (v2.0) [34]. CNV heatmap was plotted using the oncoprint function in the R package ComplexHeatmap (v2.6.2) and pheatmap. To deduce ancestry information from tumor DNA, 1000 Genomes Project phase 3 VCF release was used as our reference population [35]. Data were transformed to numeric genotypes using PLINK (v1.90b6.7). Principle component analysis (PCA) was performed using the R v3.6.0 function prcomp to establish ancestry distributions mapped by the anchor population.

Type I error (α) and type II error (β) were set at 0.05 and 0.1, respectively. Chi-square test and paired student *t*-test, as appropriate, were used to examine bivariate association of somatic differences between two cohorts. Benjamini–Hochberg was used for multiple tests. For statistical analysis and visualization, GraphPad Prism 8 was implemented (GraphPad Software, Inc.) and Rv3.6.0 packages: circlize (0.4.6), ComplexHeatmap (1.99.7), dplyr (0.8.0.1), ggplot2 (3.1.1), ggpubr (0.2), maftools (2.0.05), plyr (1.8.4), png (0.1–7), qvalue (2.16.0), reshape2 (1.4.3), stringr (1.4.0), TCGAbiolinks (2.12.6), tidyr (0.8.3), and tools (3.6.0).

## Results

### Participant characteristics

Mean age at diagnosis for all participants (*n* = 31) was 49.9 years (Table S1). Specifically, for Nigerian women (*n* = 12), mean age at diagnosis was 43.2 years old which was significantly younger than the mean age at diagnosis for Barbadian women (*n* = 19, 53.9, *p* < 0.05). For all participants with grade data (*n* = 25), 92% (*n* = 23) were diagnosed with intermediate and high grade (grade 2 or grade 3) carcinoma whereas only 8% (*n* = 2) where classified as grade 1 (Table S1). WES data were collected from The Cancer Genome Atlas (TCGA) breast cancer project, stratified by TNBC subtype and participant recorded race (African American, *n* = 24 [TCGA_TNBC_AA]; European American, n = 63 [TCGA_TNBC_EA]) for comparative analyses.

### Mutation contributions and distributions

**Fig. 1:**
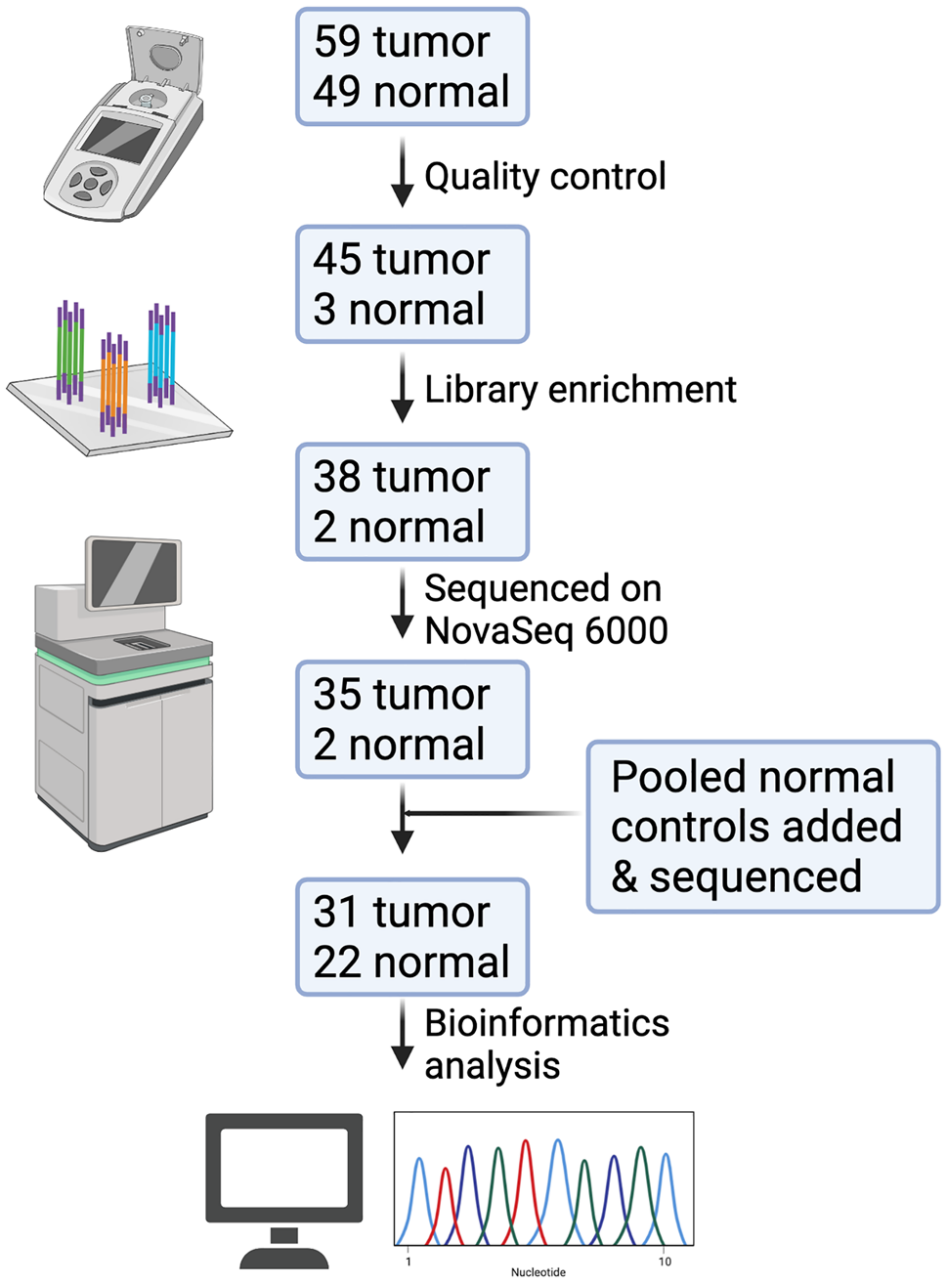
Overview of sequencing pipeline. DNA was extracted from 59 tumor-enriched samples and 49 adjacent uninvolved samples. After quality control, library enrichment and sequencing on NovaSeq6000, 31 tumor samples and 22 pooled normal adjacent controls were successfully sequenced. Model Diagram created with BioRender.com

A summary of the sequencing pipeline is depicted in Fig. 1. To assess genomic alterations in TNBC in WAA we performed WES on tumors yielding a mean output of 30,625 Mbases per sample and an average of 382 × coverage (Table S2). An internal pool approach of germline DNA samples was derived from 22 internal samples to ensure better somatic estimation instead of using available Euro-centric references [36]. Germline DNA for each sample was individually indexed before being pooled into the final capture using the same probe sets as tumor samples (Table S2). The germline pool yielded 281,792 Mbase of data and an average of 4335 × coverage showing relative germline contribution to each sample (Table S2). WES identified an average of 707 non-silent somatic mutations per tumor that was higher compared to an exome-sequenced cohort of TNBC in TCGA with a mean of 87 non-silent mutations per sample. This difference is most likely due to the residual increase in private germline variants that is contributed by the diverse African genome, as well as the larger exome capture set (this study =  ~ 80 Mbp compared to TCGA =  ~ 34 Mbp) and the internal non-tumor pool approach used for our samples. Using ancestry informative markers [37] and principal component analysis, Barbadian samples independently clustered among themselves, the African Caribbean in Barbados (ACB) group and among the Americans of African Ancestry in Southwest USA (ASW) groups (Figure S1). Nigerian samples clustered among the Yoruba in Ibadan, Nigeria (YRI) clusters and the Esan in Nigeria (ESN) clusters (Figure S1).

### Variant analysis

**Fig. 2:**
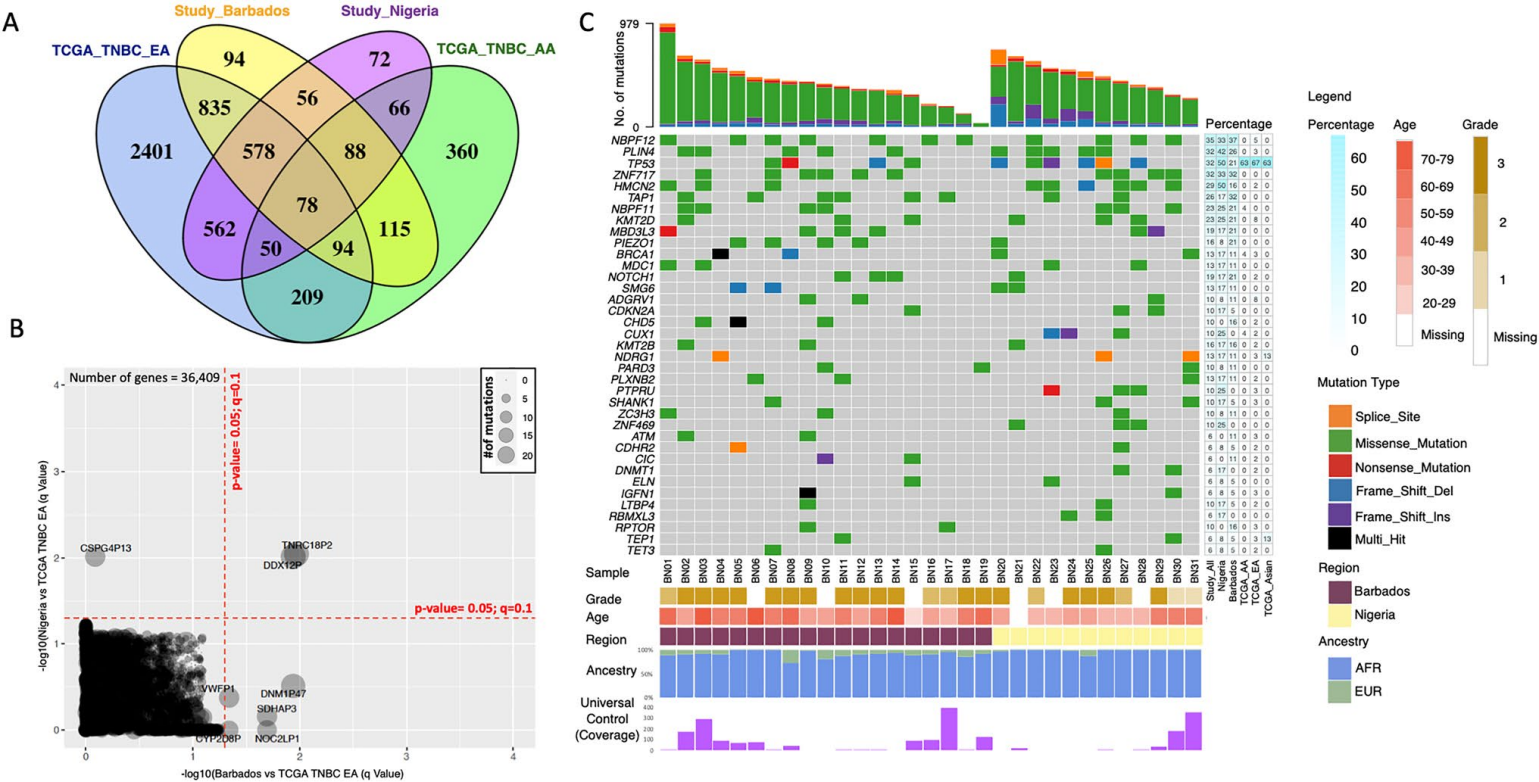
Barbadian and Nigerian women with TNBC harbour different genetic alterations than European American (EA)and African American (AA) women with TNBC. **A** Global analysis of all altered genes revealed that only 78 genes are shared among the four groups (Barbadian, Nigerian, TCGA-TNBC-EA, TCGA-TNBC-AA) and 2,401 genes are unique to the EA group in comparison to the other cohorts. **B** Global comparison of variants with TCGA-TNBC-EA group identified 2 pseudogenes (*TNRC18P2* and *DDX12P*) with an increase in variant frequency in the Nigerian (*n* = 12) and Barbadian (*n* = 19) cohorts. Benjamini–Hochberg tests used for multiple testing. **C** Genes with high frequency variants in Barbadian and Nigerian samples were not enriched in the TCGA dataset except for *TP53* (third gene from the top of the gene list)

There are 94 unique non-silent somatic variants that are enriched in Barbadian TNBC tissues (*n* = 19), and 72 unique non-silent variants enriched in Nigerian TNBC tissues (*n* = 12, Fig. 2A, Table S3–S4) where Benjamini–Hochberg tests were used for multiple testing. There were also 56 commonly mutated genes (Fig. 2A) shared between the Barbadian and Nigerian study samples and 78 commonly mutated genes (Fig. 2A) shared between the four cohorts included (Barbadian, Nigerian, TCGA-TNBC-EA/AA samples). Notably, the TCGA-TNBC-EA group had 2,401 genes in isolation that were not shared with other cohorts in our study. Global comparison of somatic variants with TCGA-TNBC-EA group (Fig. 2B) identified 2 pseudogenes that exhibited an increase in variant frequency in the Nigerian and Barbados cohort compared to TCGA-EA TNBC (*TNRC18P2* and *DDX12P*, *p* < 0.05; *q* < 0.1). However, there were no significantly mutated genes between our study samples and the TCGA-AA group (Figure S2A). Common variants in our WAA cohorts were identified in cancer-associated genes—*NBPF12*, *PLIN4, TP53, ZNF717, TAP1, KMT2D, PIEZO1* and *BRCA1* (Fig. 2C, Table S5) as well as in *HMCN2* and *MBD3L3* which have not been previously associated with cancer. In comparison with TCGA data for TNBC cases, these most frequently mutated genes in our WAA cohort were not as frequently mutated except for *TP53* (32% in our combined cohort; 63% in TCGA-TNBC-AA; 67% in TCGA-TNBC-EA; 63% in TCGA-Asian). For *TP53* specifically, there were five novel variants identified (HGVSc annotation: c.994-1_1023del, c.838_863del, c.368_374del, c.844_845insT, c.382_383del; Table S6) that were all predicted to have high impact as defined by PolyPhen2 [38].

### COSMIC mutational signatures

**Fig. 3:**
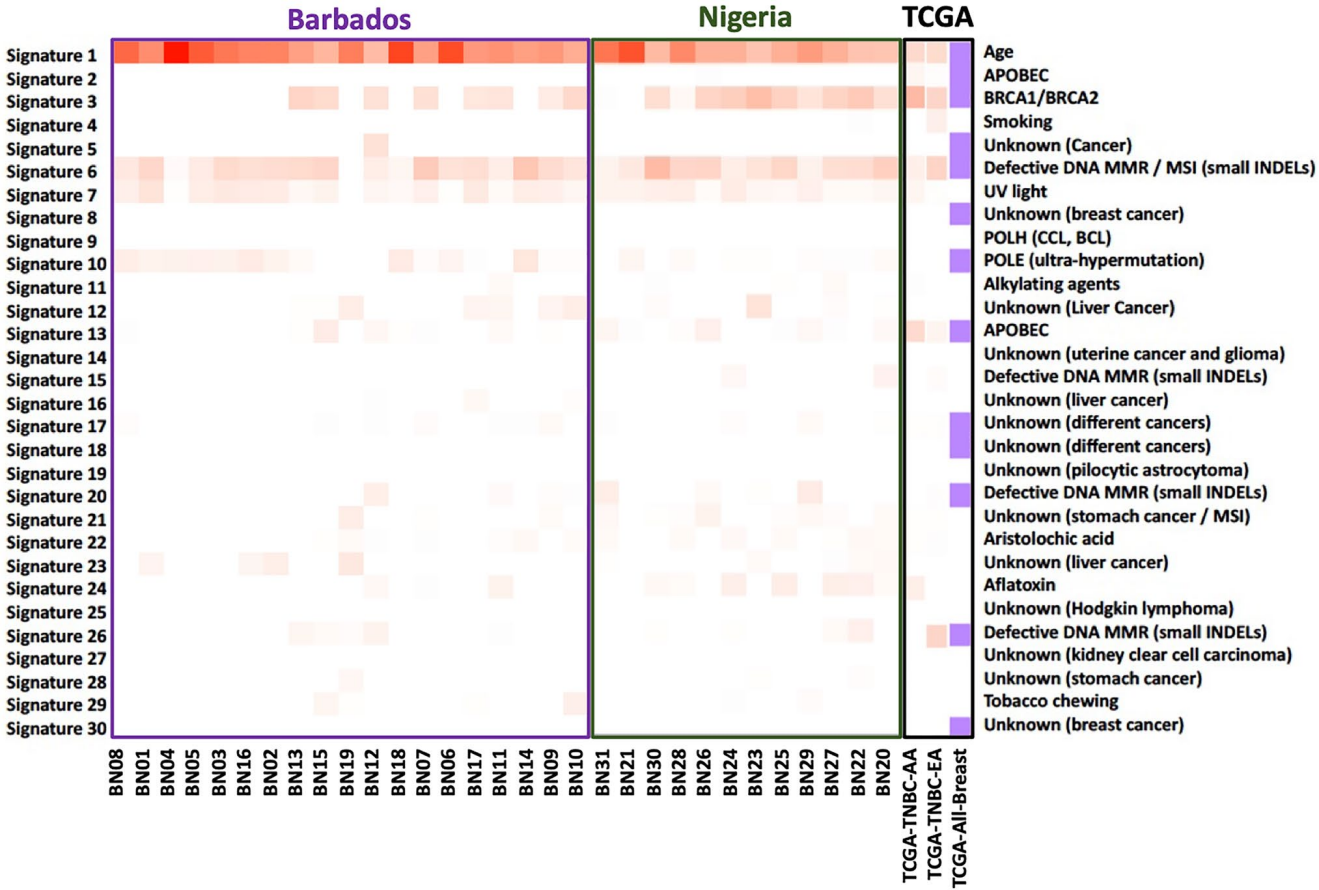
Mutation signature contributions for Barbadian and Nigerian TNBC samples show high correlation to COSMIC Signatures 1, 3 and 6. Using COSMIC somatic mutation signatures, Age, BRCA1/BRCA2 and Defective DNA MMR/MSI (small INDELS) were enriched signatures for Barbadian and Nigerian samples. These signatures were also enriched in TCGA-AA, TCGA-EA women with TNBC and overall, all breast cancer cases within TCGA

According to somatic mutation signatures as defined by COSMIC [39], most of our samples had a moderate to strong correlation with Signatures 1, 3 and 6 (Fig. 3). These signatures correspond to age, *BRCA1/BRCA2* and defective DNA mismatch repair/microsatellite instability (small INDELs), respectively. Among the Barbadian samples, there was a weak correlation with Signature 10, POLE (ultra-hypermutation), that was not seen in the Nigerian samples or the TCGA groups. There were also six Nigerian samples (54%) that showed a correlation with Signature 24 (Aflatoxin) that was not observed in the Barbadian samples or the TCGA dataset.

### Comparison of TNBC copy number variation

**Fig. 4:**
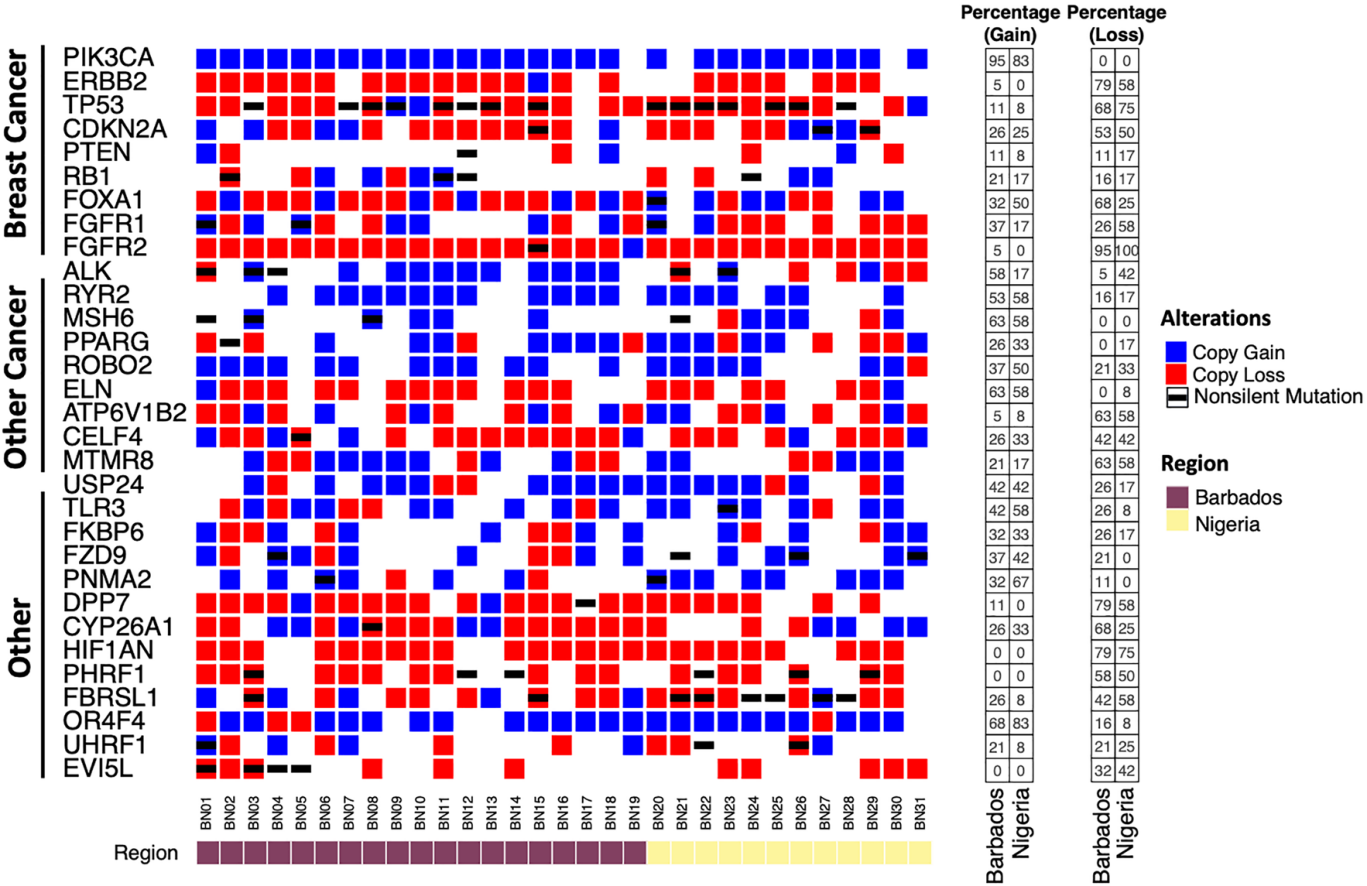
Copy number variation (CNV) analysis revealed no differences between Barbadian and Nigerian samples. Using NEXUS Copy Number (v10) analysis toolkits, most common CNV were investigated. The copy number gain across each sample is presented in blue and copy number loss in red. The black line indicates a non-silent somatic mutation. The bottom panel indicates region of sample origin. Left panels show percent alterations across each copy number event across two different populations. Bi-allelic mutation illustrated by black bar and either blue or red square indicating non-silent somatic mutation and either copy gain or less, respectively. No significance (*p* < 0.05) was detected in this cohort of samples

Copy number analysis identified several regions of the genome associated with common copy number gain and loss (Figure S3, Table S7-8). Twenty-eight out of the 31 samples (90%) harbored copy number gains in *PIK3CA,* and a copy number loss for *TP53* was seen in 23 of the 31 samples (74%). Interestingly, copy number loss was seen in 30 samples (97%) for *FGFR2* and in 24 samples (77%) for *HIF1AN*. Overall, CNVs were observed for BCa-related genes (e.g., *PIK3CA, ERBB2, TP53, FOXA1*), other cancer-related genes (e.g., *ROBO2, ELN, CELF4*) and other notable genes (e.g., *DPP7, CYP26A1*). To further expand upon our understanding of bi-allelic events across genes, an integrated analysis was performed across frequently altered genes. This analysis revealed a predominance of bi-allelic (copy number loss and non-silent mutation) in *TP53* in our study (Fig. 4).

## Discussion

Herein, we report data from WES of ancestrally related WAA (Barbadian and Nigerian) with TNBC, which revealed pathogenic and novel variants for *TP53* and *BRCA1* as well as in other BCa implicated genes such as *MDC1* (Fig. 2C) that was observed in similar BCa studies among WAA in Nigeria [20, 40]. This is in concordance with the high mutation rate for *TP53* that is typically seen in TNBC [41], and more importantly that is observed in Nigerian and African American women with TNBC [20]. Notably, we observed that 50% of our Nigerian samples (*n* = 12) harbored variants for *TP53,* which is comparable to ~ 60% of Nigerian TNBC samples (*n* = 54) with variants and copy number events observed in a previous study [20]. This suggests that variants in *TP53* might be of importance for Nigerian women with TNBC. We also observed a high frequency of variants for *NBPF12* and *PLIN4*. Interestingly, in silico analyses of BCa genomics data from TCGA and the International Cancer Genome Consortium have identified *NBPF12* as a BCa-driver gene with an estimated 0.3% substitution rate [42]. *NBPF12* belongs to the neuroblastoma breakpoint family (NBPF) of genes that are located on chromosome 1, are highly conserved across primates and are highly expressed in breast tissue [43]. *PLIN4* is located on chromosome 19 and is a member of the perilipin family that is implicated in adipocyte stability and obesity [44, 45]. Notably, high PLIN4 expression has recently been implicated in TNBC chemoresistance [46]. Although beyond the scope of this study, more functional studies need to be performed to evaluate how these genes and others identified in our study are implicated in TNBC tumorigenesis and disparity in WAA. Further studies are also needed to determine the function of the 78 genes that were commonly enriched between all study groups (Barbados, Nigeria, TCGA-AA and TCGA-EA), as they might be particularly useful for TNBC drug development regardless of ancestry.

In addition to investigating variation in individual genes, we used the COSMIC database of somatic mutations and investigated individual signatures [39], which combines base substitutions with signatures such as DNA mismatch repair. Of note, there was an enrichment of signature 24 (Aflatoxin) in 6 Nigerian samples. This signature is typically observed in a subset of hepatocellular carcinoma (HCC) liver cancers with known exposures to aflatoxin, a mycotoxin that grows on grains across West Africa and is commonly consumed among these populations [47]. It was recently documented in a 10-year study that aflatoxin contamination in crops such as maize and groundnut are common across sub-Saharan African countries such as Nigeria [48], and this may be contributing to breast and liver cancers across these sub-Saharan African nations. Two independent studies reported a considerable number of liver metastases from breast cancers in two Nigerian populations [49, 50], raising the possibility that the correlation with the aflatoxin somatic signature observed in our Nigerian TNBC cases may play a role in this phenomenon. Follow-up studies on this signature in breast tumor samples should be investigated to further delineate this relationship. Nonetheless, these findings highlight the interplay of environmental risk factors with genetics, and how they could lead to tumorigenic outcomes.

When investigating copy number variation, we observed a high enrichment of *PIK3CA* amplifications, which was also observed in previous studies [41]. Our analysis also revealed a predominance of bi-allelic (copy number loss and non-silent) mutations in *TP53* (Fig. 4) which has been previously associated with poor outcome in multiple myeloma [51]. In silico analysis of *TP53* copy number loss has also highlighted its prognostic value in breast cancer [52]. Notably, almost every sample (30/31) harboured a copy number loss at 10q26.12—q26.13 that includes the *FGFR2* gene. Multiple studies highlight an over-expression of FGFR2 and FGFR1 in TNBC [53–56] so this was an unexpected finding. Indeed, there is currently a clinical trial for inhibition of *FGFR2* (ClinicalTrials.gov identifier: NCT04526106) in solid tumors. This copy number loss of *FGFR2* highlights a novel genetic alteration in WAA not previously observed that might be protective in these populations. Further studies to investigate FGFR2 mutations and/or expression in WAA with TNBC (perhaps using different specimens—e.g., fresh frozen samples) should shed light on this phenomenon CNV data from FFPE samples can be “noisy” and CNV analyses are rapidly evolving with new tools being developed over time for better interpretation [57].

Twenty-four samples harboured a copy loss for *HIF1AN* which is the inhibitor of *HIF-1α.* In TNBC, *HIF-1α* is highly expressed and implicated in the renewal of cancer stem cells and epithelial-to-mesenchymal transition that is highly associated with metastasis [58]. The frequent copy loss of *HIF1AN* might thus be associated with the aggressive nature of TNBC observed in WAA since it prevents *HIF-1α* inhibition. Follow-up RNA sequencing, proteomic profiling and/or targeted sequencing experiments investigating the transcriptome and proteome of these WAA-TNBC cohorts will unveil genes and pathways of interest in WAA-TNBC with therapeutic implications.

It must be noted that social determinants of health (SDoH) should also be considered as factors in our findings since genomics testing is not routinely available among these populations. Though the costs of genomics testing are decreasing, these tests are not as accessible to resource-limited settings for routine clinical (diagnostic and prognostic) tests due to lack of infrastructure [59]. Most recent analyses from GLOBOCAN, estimated the highest BCa mortality rates in countries across West Africa and the Caribbean (with Barbados having the world’s highest mortality) [60]. This high mortality rate in Barbados might be explained by the high incidence of biologically aggressive tumours and advanced staged tumours as we previously reported [4, 61]. Indeed, a recent study investigating approaches to cancer control across the Caribbean region highlighted that there was no organised BCa national screening programme present in Barbados [62]. This might be due to lack of resources and the use of mammography services primarily for diagnostic measures rather than screening purposes. A similar scenario was observed across West African countries where financial constraints and belief in traditional medicine were contributing factors to overall BCa burden [63, 64]. Therefore, to fully understand BCa etiology and progression in these and other underrepresented populations, SDoH should be taken into consideration when highlighting genomic alterations.

### Limitations

These data are specific to the samples that were included in the study, and not generalizable to all Barbadian and Nigerian women with TNBC. Further follow-up studies with more samples, appropriate germline controls and associated clinical data are needed to identify potential biomarkers and clinical utility among these populations. It should also be noted that tumor microenvironment as well as inter- and intra- tumour heterogeneity are factors that can be impacted by the sections of the tumor and non-tumor sections that were sequenced and can therefore affect variants called and any potential clinical relevance [65]. To differentiate between somatic and germline mutations, genomic DNA is routinely extracted from peripheral blood, saliva, and adjacent healthy tissue representing germline spectrum of genomics data. This however was not possible due to our retrospective study design as well as the limited resource settings in Barbados and Nigeria at the time of data collection. We acknowledge this limitation and created a pooled non-tumor sample derived from the low quantity patient’s germline adjacent normal breast tissue. This was the best approach instead of only relying on the current Euro-centric databases for germline subtraction with low representation of African ancestry genomics data [36]. We also performed a manual IGV [31] review of each highlighted variant presented in Fig. 2C to remove any false positives due to the higher potential of inflation in false positive somatic calling. We acknowledge that there may still be infiltration from naturally occurring polymorphisms in our variant calls due to not having matched tumor/normal controls. We initially used ExAC and 1000 Genomes databases for variant calls and this resulted in ~ 2 to 3-fold enrichment of potential population related germline false positives in our somatic calling when compared to our pooled reference approach (data not shown). We are confident that our pooled reference approach was appropriate given these challenges. However, further functional validation is required to assess these variant calls and associated clinical relevance. To address other issues with extracting high quality nucleic acids derived from FFPE tissues, we sequenced our tumor and non-tumor samples at high-resolution sequencing depth (382 × and 4,335 × , respectively) to increase confidence in our variant calls. Our pooled germline sample approach might be a useful application for studies of solid tumors with limited germline availability in other resource-limited populations/healthcare facilities.

## Conclusions

To our knowledge this is the first study to investigate the mutational landscape of TNBC patients from populations with related African ancestry—West Africa (Nigeria) and Caribbean (Barbados). We identified pathogenic and likely pathogenic variants and novel variants in cancer-associated genes (e.g., *TP53*, *BRCA1*, *MDC1*) and variants in other potential genes of interest (e.g., *NBPF12*, *PLIN4*, *FGFR2*). These variants may be useful for development of future therapeutic options, both unique to our WAA-TNBC cohorts and universally for all women diagnosed with TNBC. Furthermore, to better reflect our global population, more collaborative studies need to be done to increase genomic data from diverse populations within genomics databases. This would allow researchers to identify genetic risks and therapeutic options for diverse populations.

## Supplementary Information

Below is the link to the electronic supplementary material.

Supplementary file1 (PNG 494 kb)

Supplementary file2 (PNG 229 kb)

Supplementary file3 (PNG 325 kb)

Supplementary file4 (XLSX 243 kb)

## Data Availability

The manuscript has electronic data included as electronic supplementary material. Raw sequence data will be submitted to NCBI SRA upon acceptance of the manuscript.
