# Supplementary figures and images for: Analysis of the genomic landscapes of Barbadian and Nigerian women with triple negative breast cancer
Source: Cancer Causes Control. 2022 Apr 6;33(6):831–41. doi: 10.1007/s10552-022-01574-x (PMC9085672; doi:10.1007/s10552-022-01574-x)

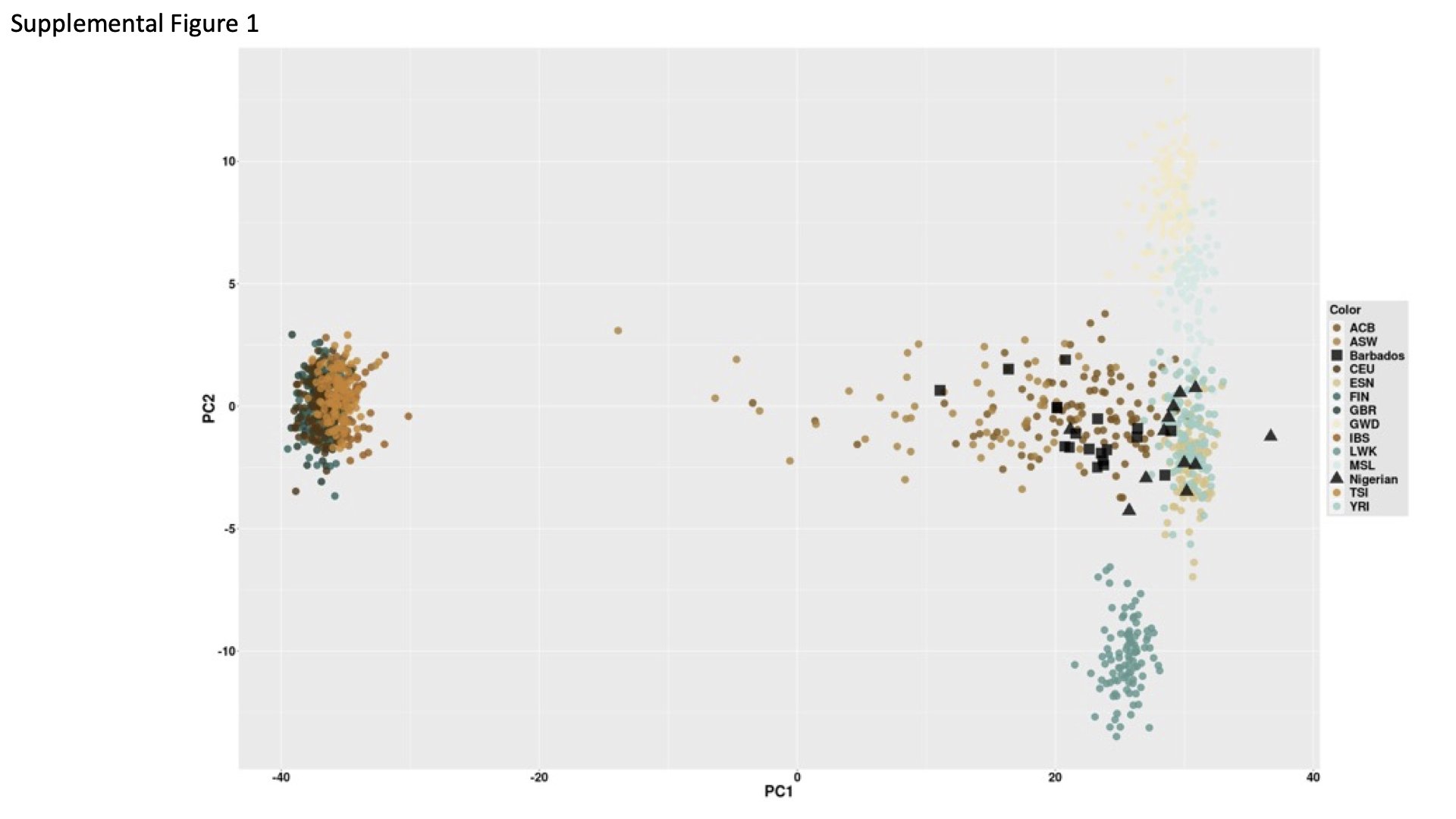

Supplement: Supplementary file 1 — Supplementary file1 (PNG 494 kb) [file 10552_2022_1574_MOESM1_ESM.png]

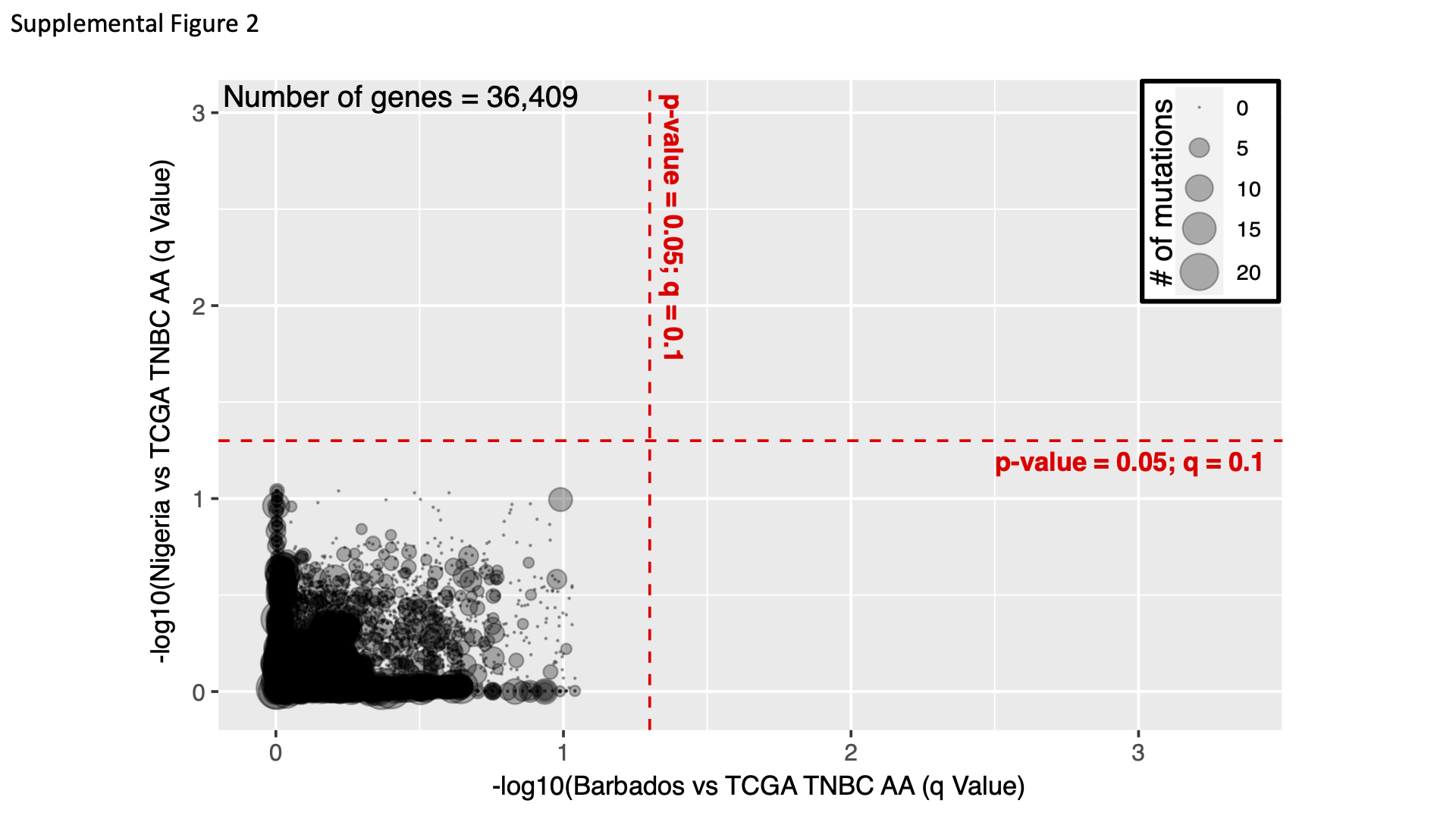

Supplement: Supplementary file 2 — Supplementary file2 (PNG 229 kb) [file 10552_2022_1574_MOESM2_ESM.png]

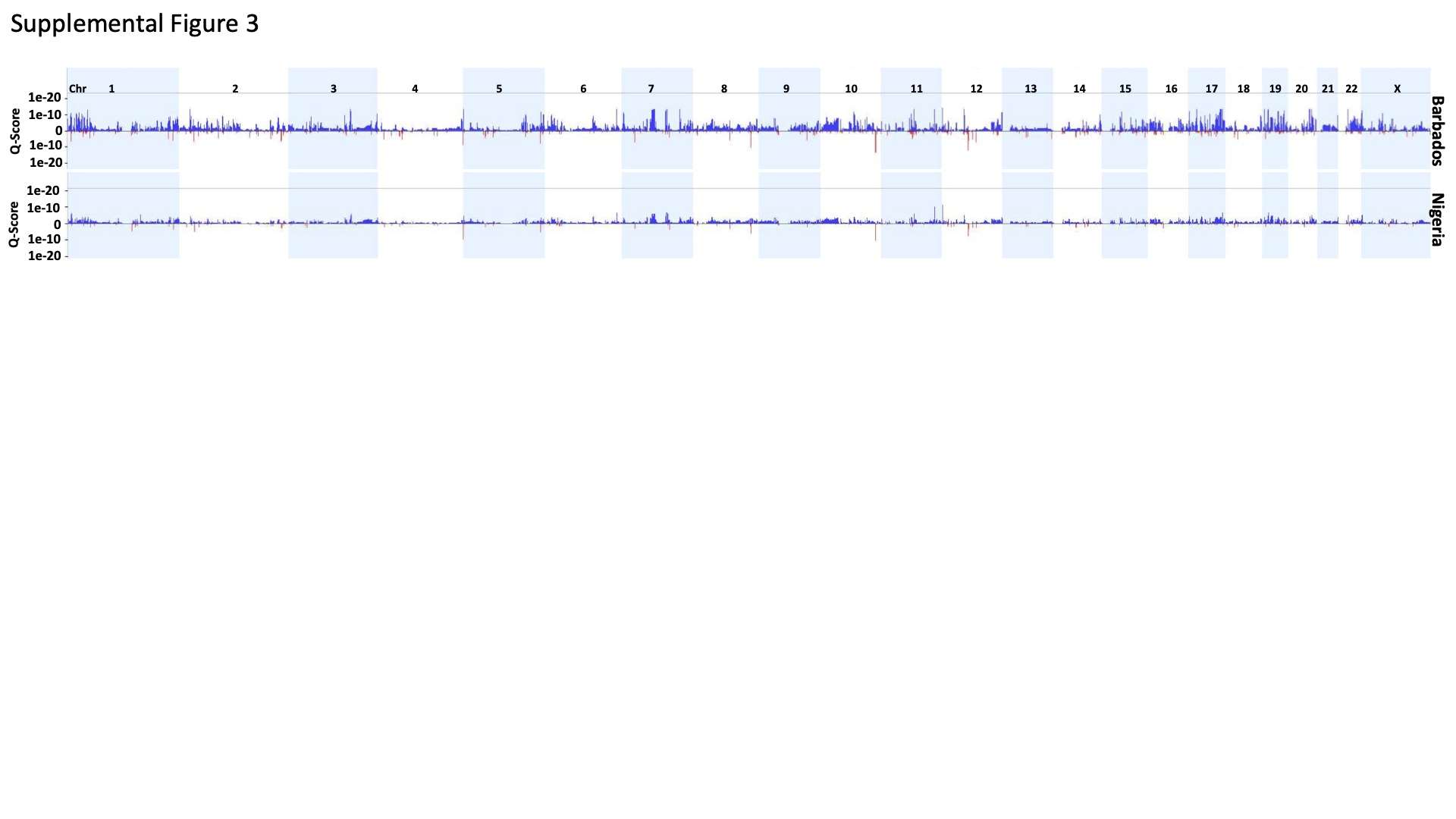

Supplement: Supplementary file 3 — Supplementary file3 (PNG 325 kb) [file 10552_2022_1574_MOESM3_ESM.png]
